# Hydroxytyrosol Prevents Increase of Osteoarthritis Markers in Human Chondrocytes Treated with Hydrogen Peroxide or Growth-Related Oncogene α

**DOI:** 10.1371/journal.pone.0109724

**Published:** 2014-10-03

**Authors:** Annalisa Facchini, Silvia Cetrullo, Stefania D'Adamo, Serena Guidotti, Manuela Minguzzi, Andrea Facchini, Rosa Maria Borzì, Flavio Flamigni

**Affiliations:** 1 Dipartimento di Scienze Mediche e Chirurgiche, Università di Bologna, Bologna, Italy; 2 Dipartimento di Scienze Biomediche e Neuromotorie, Università di Bologna, Bologna, Italy; 3 Laboratorio di Immunoreumatologia and Rigenerazione Tissutale/Laboratorio RAMSES, Istituti Ortopedici Rizzoli, Bologna, Italy; SERGAS, Santiago University Clinical Hospital, IDIS Research Laboratory 9, NEIRID Lab, Spain

## Abstract

Hydroxytyrosol (HT), a phenolic compound mainly derived from olives, has been proposed as a nutraceutical useful in prevention or treatment of degenerative diseases. In the present study we have evaluated the ability of HT to counteract the appearance of osteoarthritis (OA) features in human chondrocytes. Pre-treatment of monolayer cultures of chondrocytes with HT was effective in preventing accumulation of reactive oxidant species (ROS), DNA damage and cell death induced by H_2_O_2_ exposure, as well as the increase in the mRNA level of pro-inflammatory, matrix-degrading and hypertrophy marker genes, such as iNOS, COX-2, MMP-13, RUNX-2 and VEGF. HT alone slightly enhanced ROS production, but did not enhance cell damage and death or the expression of OA-related genes. Moreover HT was tested in an *in vitro* model of OA, i.e. three-dimensional micromass cultures of chondrocytes stimulated with growth-related oncogene α (GROα), a chemokine involved in OA pathogenesis and known to promote hypertrophy and terminal differentiation of chondrocytes. In micromass constructs, HT pre-treatment inhibited the increases in caspase activity and the level of the messengers for iNOS, COX-2, MMP-13, RUNX-2 and VEGF elicited by GROα. In addition, HT significantly increased the level of SIRT-1 mRNA in the presence of GROα. In conclusion, the present study shows that HT reduces oxidative stress and damage, exerts pro-survival and anti-apoptotic actions and favourably influences the expression of critical OA-related genes in human chondrocytes treated with stressors promoting OA-like features.

## Introduction

Chondrocytes, the only cell type in adult cartilage, are usually kept in a quiescent, maturation-arrested state, and maintain tissue integrity by a low turnover of extra-cellular matrix (ECM) components. However in osteoarthritis (OA), a common chronic degenerative- and ageing-associated disease, a disorganized recapitulation of endochondral ossification is promoted, leading to hypertrophic differentiation and apoptosis of chondrocytes, associated to ECM degradation and mineralization [1]. Among key molecular effectors driving these processes are runt-related transcription factor 2 (RUNX-2), matrix metalloproteinase-13 (MMP-13) and the angiogenic vascular endothelial growth factor (VEGF). In the context of OA, chondrocytes produce pro-inflammatory agents, such as cytokines, chemokines, eicosanoids (e.g., PGE_2_) and nitric oxide (NO), as well as an array of hydrolytic enzymes, which in an autocrine/paracrine manner contribute to terminal differentiation of chondrocytes and ECM degradation [2]–[4]. Moreover in response to mechanical, inflammatory and metabolic stressors, chondrocytes become both source and target of elevated amounts of reactive chemical species, particularly oxygen- and nitrogen-species, which cause oxidative stress, thus establishing positive feed-back loops and resulting in further damage of cartilage cells and matrix [5].

An effective and safe strategy for OA prevention and therapy is still lacking. Pharmacological treatments nowadays available, mainly non-steroidal anti-inflammatory drugs (NSAID), do not affect OA progression substantially and present disadvantages, such as side effects and high cost. Therefore the search for molecules able to interfere with molecular mechanisms of OA pathogenesis represents an important challenge [6]. In particular several dietary factors and nutraceuticals are promising [7], [8], but extensive investigation in preclinical and clinical settings is required to prove their usefulness. On this purpose, we and others have showed the ability of sulforaphane, a natural isothiocyanate derived from edible cruciferous vegetables, to protect chondrocytes *in vitro* [9]–[11], and, quite recently, a sulforaphane-rich diet was found effective in a murine OA model [12]. Another interesting candidate molecule is hydroxytyrosol (HT), a phenolic compound endowed of a powerful anti-oxidant action, mainly found in the fruits of olive tree (*Olea europaea* L.) and their derivatives, such as olive oil [13]. Several studies, mostly performed in cell and animal models, have revealed a range of biological properties of HT, suggesting beneficial effects in the prevention or treatment of chronic and degenerative diseases, especially cardiovascular disease and cancer. In particular HT has been shown to display cytoprotective and anti-inflammatory actions in a variety of cell types [14]–[23]. However, to our knowledge, information is lacking about the effects of HT on chondrocytes and cartilage. In the present study, we report data about the action of HT in monolayer and tridimensional cultures of human chondrocytes, showing that HT afforded protection against chondrocyte damage, apoptosis and expression of OA-related markers.

## Methods

### Ethics Statement

Preclinical research involving human OA patient cartilage tissue samples at the Rizzoli Orthopaedic Institute was subjected to the approval of the ethics committee/institutional review board of the Institute (“Comitato Etico dell’Istituto Ortopedico Rizzoli”), which included documentation of written patient consent forms. Prior to the retrieval of tissues from surgeons, all patient identifiers were removed from tissue samples which were coded by arbitrary designations to distinguish them solely for experimental purposes.

### Cell culture and treatment

With local Ethics Committee approval, primary cultures of chondrocytes were used and prepared from fragments of articular cartilage obtained from 23 adult OA patients (age 63–83) undergoing knee arthroplasty as described [24]. Chondrocytes were cultured in D-MEM and 10% FCS as previously detailed [24]. For experiments in monolayer, chondrocytes were incubated in the absence or presence of 100 µM H_2_O_2_ for 1 to 48 h as indicated in the various figures; 100 µM HT (from Sigma Chemical Company, St. Louis, MO, or Cayman chemical, MI, USA, dissolved in DMSO or ethanol) was added 30 min before H_2_O_2_. Cells not pre-treated with HT received equal amount of the vehicle. The concentration of HT was chosen on the basis of a published study [25] and preliminary experiments in a human chondrocyte cell line. Viable cells were directly counted following the trypan blue exclusion test. Dead cells were also counted and reported as a percentage of the total number of cells. Care was taken to recover even possible detached cells. OA chondrocytes were also seeded into micromass cultures as previously described [26]. The medium was changed every second day and the micromasses were used after 1, 2 or 3 weeks of culture. Before stimulation, the micromasses were kept in 500 µl 3% FCS DMEM for 24 hours and volumes were reduced to 100 µl when 100 nM growth-related oncogene α (GROα) (R&D systems, Minneapolis, MN) was added for 90 min; 100 µM HT was added 30 min before GROα.

### Detection of reactive oxidant species

Reactive oxidant species (ROS) were detected in monolayer cultures of chondrocytes (5,000 cells per well in a 96 wells cluster) by using the oxidant-sensitive probe 2′, 7′-dichlorofluorescein diacetate (DCF-DA) (Sigma) that emits green fluorescence when oxidized [27]. The cells were incubated with 30 µM DCF-DA for 30 min at 37°C in complete medium. After change of medium to remove DCF-DA, the cells were incubated with HT, H_2_O_2_ or GROα for the established time. Then the cells were detached from the wells with trypsin, centrifuged and finally resuspended in PBS. Thereafter, the production of ROS was measured in the cells by using a WallacVictor2 1420 Multilabel Counter fluorimeter, at the wavelength of excitation of 485 nm and emission of 538 nm. Measures of samples were corrected by subtracting a proper blank, given by basal fluorescence intensity of cells incubated without DCF-DA. Measures of fluorescence were also performed in the conditioned cell medium that was collected at the end of incubation, and corrected by subtracting the fluorescence of non-conditioned medium incubated with DCF-DA.

### Detection of γH2AX

Flow cytometry was employed to evaluate the extent of oxidative DNA damage and in particular the amount of double strand breaks tagged by the phosphorylation of the histone H2AX in the areas surrounding the lesions. Immunostaining for flow cytometry was performed using cells that had been previously fixed with 2% PFA and then post-fixed with 90% methanol on ice. The first step of “antigen unmasking” was carried out with 0.02 U/ml chondroitinase ABC in Tris HCL pH 8 (20′, 37°C) to increase the permeability of the cells to the antibodies. A blocking step was performed before immunostaining, using TBS supplemented with 5% bovine serum albumin (BSA) and 0.1% Triton, left for 30′ at RT. Primary and secondary antibody dilutions were done in TBS supplemented by 3% BSA and 0.1% Triton. To detect double strand breaks marked by phosphorylation of histone H2AX, we used a mouse anti-γH2AX monoclonal antibody (Millipore 05–636, IgG1, at 5 µg/ml) incubated overnight at 4°C. The secondary antibody, used at 15 µg/ml, was Alexa Fluor 647 Donkey anti mouse IgG (Jackson Labs) and was incubated for 30 min at RT. Specificity was checked by running an isotypic control for each sample under analysis: cells were probed with normal mouse immunoglobulins of the same isotype (IgG1) and at the same concentration of the primary antibody anti-γH2AX. Analyses were performed using an FACS Canto II flow cytometer (BD). At least 5000 cells were analyzed for each tube and distribution of fluorescence intensity was recorded. For each sample under analysis the level of “median channel of fluorescence intensity (MCFI) increment” of γ-H2AX staining was calculated as the difference between the median channel of fluorescence intensity of the samples stained for γH2AX and that of the same sample probed with the isotypic control.

### Determination of caspase activity

Caspase activity was measured by the cleavage of the fluorogenic peptide substrate Ac-Asp-Glu-Val-Asp-7-amido-4-methylcoumarin (Ac-DEVD-AMC) as previously detailed [24]. Since the sequence DEVD represents a substrate for caspase-3 and other effector caspases, this activity has been referred to as caspase 3-like activity. Caspase activity was expressed per mg protein, and normalized to untreated controls.

### Quantitative Real Time PCR (qRT-PCR) analysis

The micromasses were kept frozen at −20°C after treatment and then, like chondrocytes grown in monolayer, extracted with 300 µl TRIZOL (Invitrogen), following manufacturer’s instructions. The RNA pellets were treated with DNAse (DNA-free, Ambion, Austin, TX) and after buffer exchange and concentration to a 5 µl volume the RNA was reverse transcribed with oligo-dT and Superscript First-Strand Synthesis System for RT-PCR (Invitrogen), according to manufacturer’s instructions. cDNA was quantified by means of the PicoGreen double-stranded DNA quantitation reagent (Molecular Probes, Eugene OR) and then diluted to the same concentration (5 ng/ml), in order to exploit the same range of amplification efficiency. Real time PCR analysis was run in a LightCycler Instrument (Roche Molecular Biochemicals) by means of the QuantiTect SYBR Green PCR kit (TaKaRa, Japan) with the following protocol: initial activation of HotStart Taq DNA polymerase at 95°C for 10″, followed by amplification (40 cycles: 95°C for 5″ followed by appropriate annealing temperature for each target as detailed below kept for 20″). The protocol was concluded by melting curve analysis to check amplicon specificity. Two microliters of each sample was processed for each gene under study. The following primers (from Invitrogen) were used: COX-2 (NM_000963.3) 702-722F and 812-830R; GAPDH (NM_002046) 579–598F and 701-683R; iNOS (NM_000625.4) 2183-2205F and 2398-2418R; MMP-13 (NM_002427) 496–511F and 772-756R; RUNX-2 variant transcript 3 (NM_004348) 864–883F and 968-949R, RUNX-2 variant transcripts 2 (NM_001015051) and 1 (NM_001024630) 716–735F and 820-801R; SIRT-1 (NM_0012238.4) 733-755F and 891-912R; VEGF variant transcripts7 (NM_001033756.1), 6 (NM_001025370.1), 5 (NM_001025369.1), 4 (NM_001025368.1), 3 (NM_001025367.1), 2 (NM_003376.4), and 1 (NM_001025366.1), 1144-1126 (forward) and 1063–1079 (reverse). Primers were annealed at 56°C, except COX-2 at 60°C, SIRT-1 and iNOS at 58°C. The amount of mRNA was normalized for GAPDH expression in each sample and referred to untreated, control sample.

### Statistical Analysis

All data shown in graphs are expressed as means ± S.E.M of *n* separate determinations. Means of groups were compared with GraphPad Prism 5 statistical software (GraphPad Software, Inc.) and analysed for statistical significance (* = *p* < 0.05, ** = *p* < 0.01, *** = *p* < 0.001) by two-way ANOVA followed by Bonferroni post-hoc tests.

## Results

### Effects of hydroxytyrosol on human chondrocytes treated with hydrogen peroxide

**Figure 1:**
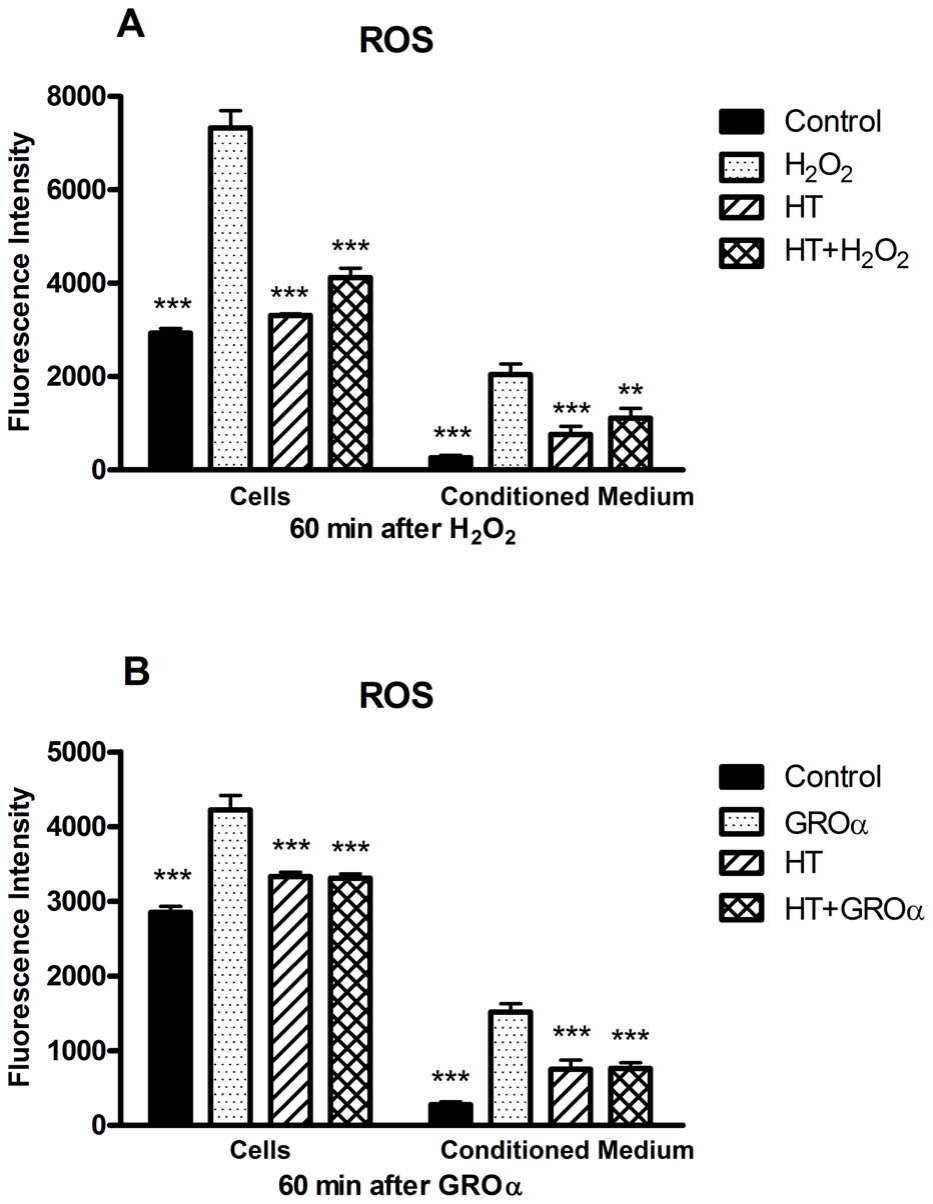
Hydroxytyrosol reduces the levels of ROS in chondrocytes treated with H_2_O_2_ or the chemokine GROα. Monolayer cultures of OA chondrocytes, loaded with the fluorogenic probe DCF-DA as detailed in Methods, were pre-incubated in the absence or in the presence of HT for 30 min before addition of H_2_O_2_ (**A**) or GROα (**B**). After 60 min, cells and conditioned medium were separated and assayed for reactive chemical species. Data are means ± S.E.M. of 6 (A) or 4 (B) separate determinations; ** *p*<0.01 and *** *p*<0.001 vs. cells treated only with H_2_O_2_ or GROα. Interestingly, comparison of all control cells and cells treated only with HT (A+B) showed a significant difference between means (*p*<0.05).

**Figure 2:**
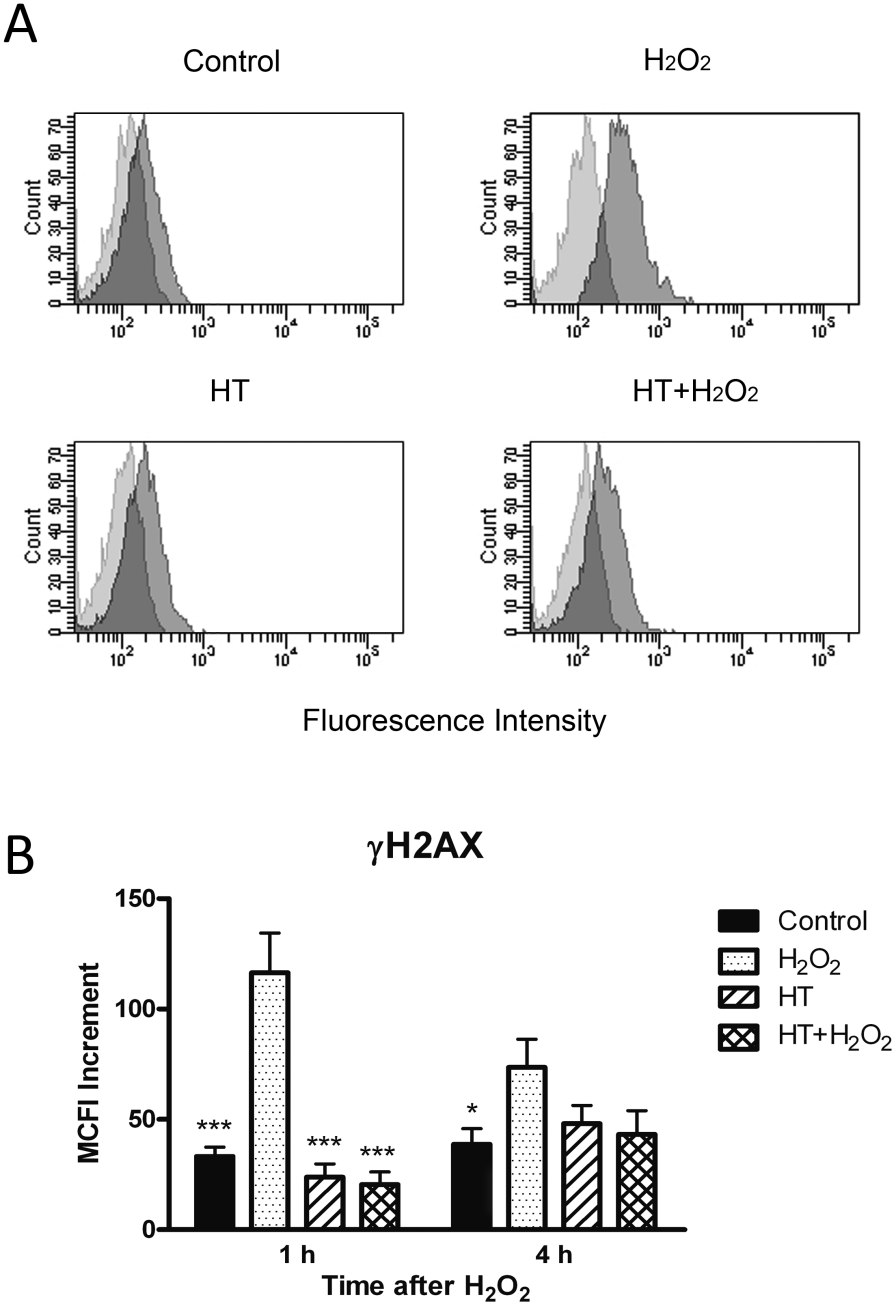
Hydroxytyrosol prevents DNA damage in chondrocytes treated with H_2_O_2_. Monolayer cultures of OA chondrocytes, pre-treated with or without HT for 30 min, were incubated with H_2_O_2_ for 1 h or 4 h. Chondrocytes were then evaluated for the extent of DNA damage by detection of γH2AX. In (**A**) a flow cytometric analysis of γH2AX of representative samples from cells incubated for 1 h is shown. Light grey histograms represent isotype control and dark grey histograms represent γH2AX-specific antibodies. In (**B**) the increment of the mean channel fluorescence intensity (MCFI) over basal fluorescence was calculated for all samples analyzed by flow cytometry and cumulative results from 1 h and 4 h incubation experiments are shown. Data are means ± S.E.M. of 5 (1 h) or 6 (4 h) separate determinations; * *p*<0.05 and *** *p*<0.001 vs. cells treated only with H_2_O_2_.

Articular chondrocytes from OA patients were cultured in monolayer and treated with H_2_O_2_ to induce oxidative stress. The level of reactive chemical species in cells was determined by the assay with the oxidant-sensitive probe DCF-DA. Exposure to H_2_O_2_ for 1 h resulted in a 2.5 fold increase of fluorescence intensity with respect to control, untreated cells. HT enhanced the intensity of fluorescence only slightly, but markedly reduced the increase induced by H_2_O_2_ (Figure 1A). In conditioned medium reactive chemicals were detected at lower levels, but likewise modulated by H_2_O_2_ and HT, suggesting that HT may quench even the accumulation of extra-cellular ROS. Figure 2A shows how the oxidative stress elicited a notable DNA damage in cells after 1 h as indicated by increased detection of a phosphorylated form of histone H2AX referred to as γH2AX, a widely used marker of DNA double strand breaks. HT alone did not affect significantly the extent of DNA damage, but completely prevented the increase by H_2_O_2_. It should be noted that the DNA damage was attenuated after 4 h treatment with H_2_O_2_ indicating the occurrence of DNA repair (Figure 2B) [28].

**Figure 3:**
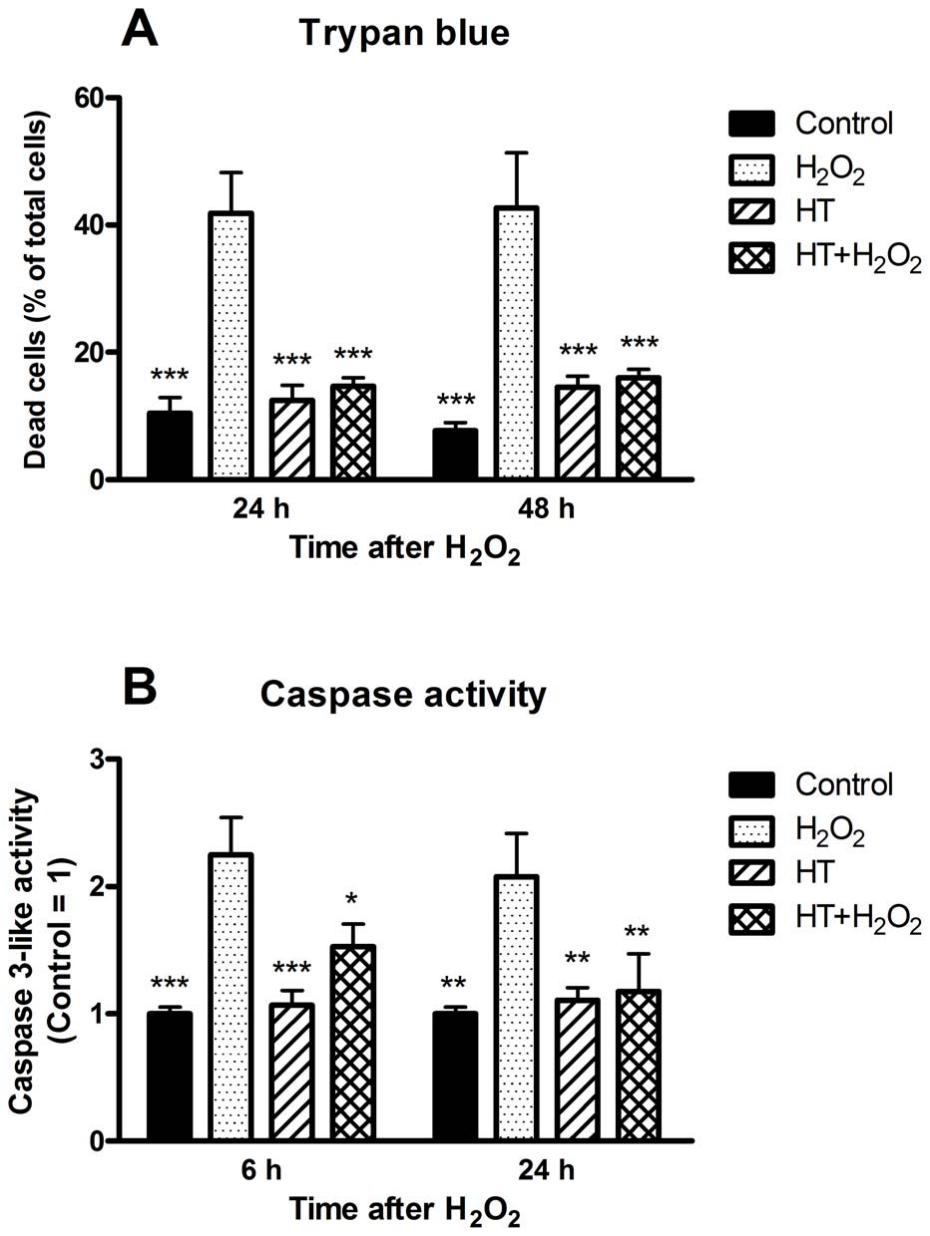
Cytoprotective action of hydroxytyrosol on chondrocytes treated with H_2_O_2_. Monolayer cultures of OA chondrocytes were pre-incubated in the absence or in the presence of HT for 30 min before addition of H_2_O_2_. At the time indicated after H_2_O_2_ cells were counted to assess cell viability by trypan blue exclusion test (**A**) or analysed for caspase activity (**B**). Data are means ± S.E.M. of 6 separate determinations; * *p*<0.05, ** *p*<0.01 and *** *p*<0.001 vs. cells treated only with H_2_O_2_. Other comparisons between means showed not significant differences.

However, H_2_O_2_ provoked a significant increase in the percentage of dead cells estimated by trypan blue exclusion test after 24 h and 48 h of treatment (Figure 3A). Exposure of cell cultures to HT did not affect cell viability significantly by itself, but prevented the enhancement of cell death caused by H_2_O_2_. The H_2_O_2_-elicited enhancement in cell death was preceded and accompanied by a significant, albeit limited, increase of caspase 3-like activity, which was likewise prevented by HT pre-treatment (Figure 3B). Therefore caspase-dependent apoptosis can partially contribute to cell death induced by H_2_O_2_ treatment, but HT afforded cytoprotection regardless of the types of cell death that were involved.

**Figure 4:**
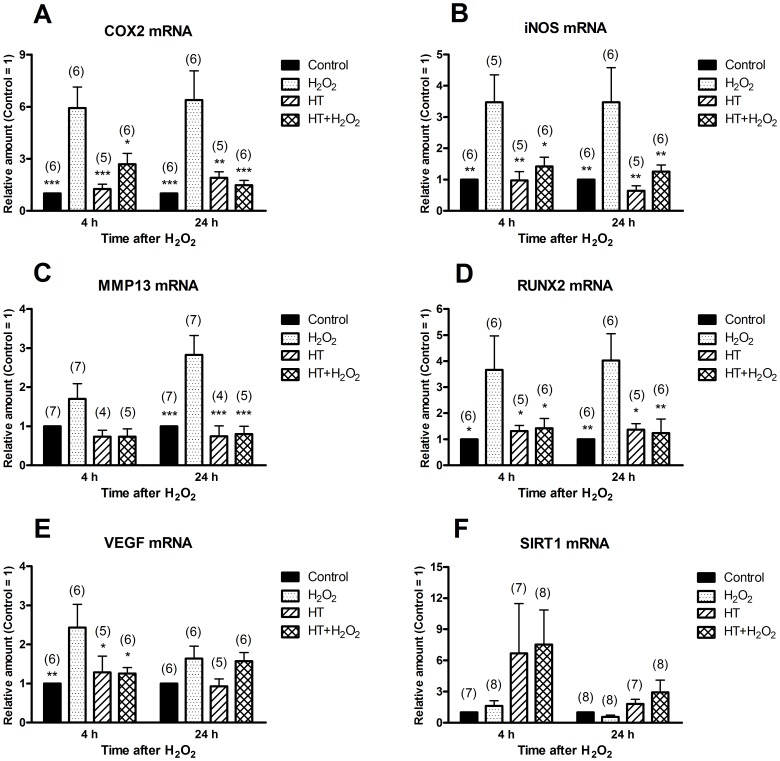
Hydroxytyrosol prevents the expression of OA-related genes in chondrocytes treated with H_2_O_2_. Monolayer cultures of OA chondrocytes were pre-incubated in the absence or in the presence of HT for 30 min before addition of H_2_O_2_. At the time indicated after H_2_O_2_, cells were harvested and analysed by qRT-PCR for the amount of COX-2 mRNA (**A**), iNOS mRNA (**B**), MMP-13 mRNA (**C**), RUNX-2 mRNA (**D**), VEGF mRNA (**E**) and SIRT-1 mRNA (F). Data are means ± S.E.M. of (*n*) separate determinations; * *p*<0.05, ** *p*<0.01 and *** *p*<0.001 vs. cells treated only with H_2_O_2_. Other comparisons between means showed not significant differences.

The effect of H_2_O_2_ treatment on the expression of a selected subset of genes known to be involved in OA pathogenesis was investigated by qRT-PCR. Figure 4 shows that H_2_O_2_ significantly increased mRNA levels of COX-2, iNOS, MMP-13, RUNX-2 and VEGF after 4 h and/or 24 h of treatment. Addition of HT alone to cell cultures did not affect the expression of these genes with respect to control cells significantly, however HT was able to prevent or reduce the increases induced by H_2_O_2_. We have also considered SIRT-1, which may exert a pivotal role in protecting from OA according to recent evidence (reviewed in [29]), but the levels of SIRT-1 mRNA did not vary significantly following treatments with H_2_O_2_ and/or HT (Figure 4F).

### Effects of hydroxytyrosol on human chondrocytes treated with growth-related oncogene α

Growth-related oncogene α (GROα) is a CXC chemokine particularly abundant in the OA chondrocyte inflammatory environment and can play an important role in cartilage derangement [3]. The chemokine was able to enhance the production of ROS by 50% in monolayer cultures of primary chondrocytes, an effect reduced by HT pretreatment (Figure 1B). However, we have previously shown that chondrocyte hypertrophy and apoptosis induced by GROα require three-dimensional interaction with ECM as revealed in 3-D micromass cultures of chondrocytes, but not in usual monolayer cultures [26]. Therefore experiments were also performed with micromass cultures, which more faithfully reflect the *in vivo* situation.

**Figure 5:**
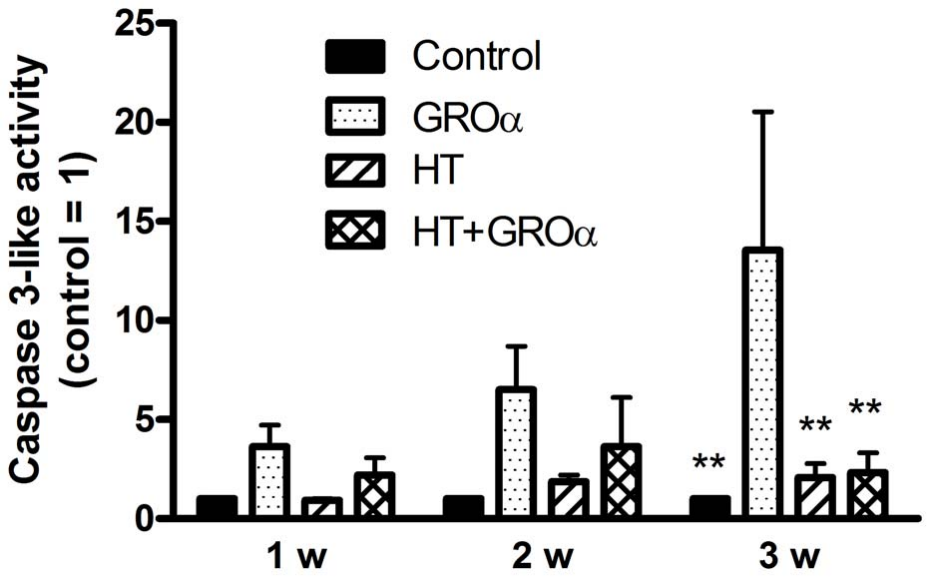
Hydroxytyrosol inhibits the increase of caspase activity in 3-D cultures of chondrocytes treated with GROα. OA chondrocyte micromasses at 1, 2 and 3 weeks (w) of maturation were pre-incubated in the absence or in the presence of HT for 30 min before addition of GROα as described in Methods. Then micromasses were harvested and analysed for caspase activity. Data are means ± S.E.M. of 5 separate determinations; ** *p*<0.01 vs. cells treated only with GROα. Other comparisons between means showed not significant differences.

GROα was able to provoke an increase of effector caspase 3-like activity, as previously shown [9]. However the sensitivity to this effect of the chemokine appeared to increase with the maturation stage of the 3-D cultures reaching statistical significance only with 3 weeks-old micromasses (Figure 5). Noteworthy, 1 week-old micromasses proved to be more resistant to apoptotic stimuli compared to 3 week micromasses, consistently with the higher amount of collagen 2, which represents a ligand for receptor-mediated cell-matrix interactions, thus mediating FAK-dependent cell survival signalling as demonstrated in mice lacking collagen II [30].

**Figure 6:**
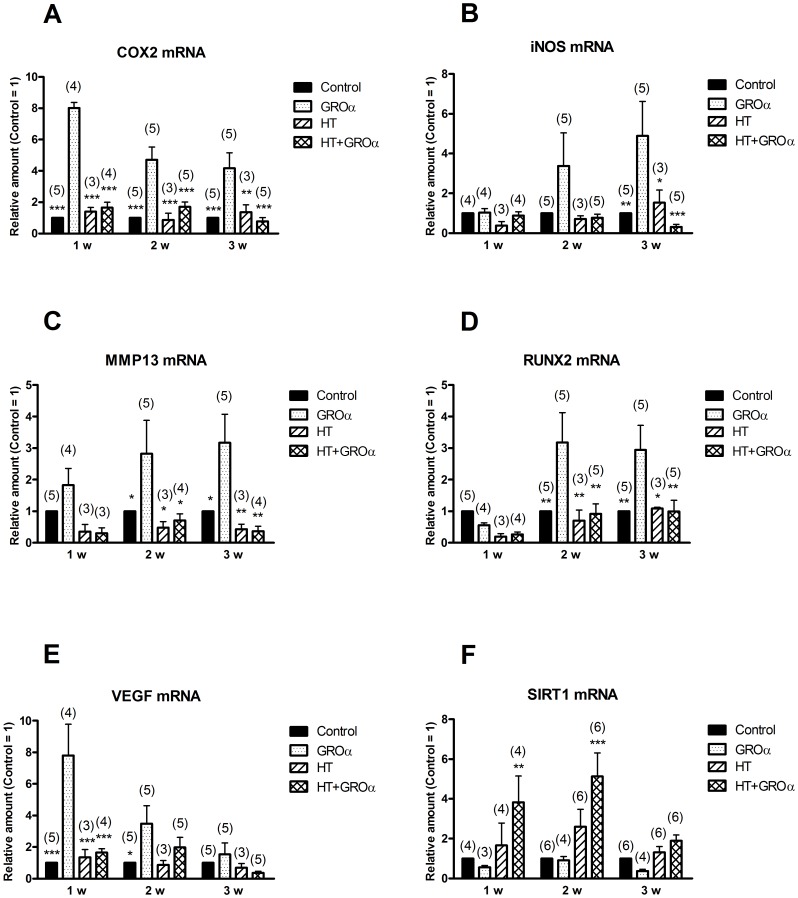
Hydroxytyrosol regulates the expression of OA-related genes in 3-D cultures of chondrocytes treated with GROα. OA chondrocyte micromasses at 1, 2 and 3 weeks maturation were pre-incubated in the absence or in the presence of HT for 30 min before addition of GROα as described in Methods. Then micromasses were harvested and analysed by qRT-PCR for the amount of COX-2 mRNA (**A**), iNOS mRNA (**B**), MMP-13 mRNA (**C**), RUNX-2 mRNA (**D**), VEGF mRNA (**E**) and SIRT-1 mRNA (**F**). Data are means ± S.E.M. of (*n*) separate determinations; * *p*<0.05, ** *p*<0.01 and *** *p*<0.001 vs. cells treated only with GROα. Other comparisons between means showed not significant differences, except for Control vs. HT+GROα at 1 w (* *p*<0.05) and at 2 w (*** *p*<0.001) in panel F (SIRT-1 mRNA).

Next, the expression of OA-related genes following GROα treatment of 1-, 2- and 3-weeks old micromasses was examined together with the HT effect (Figure 6). Panels A–E show that GROα was able to enhance the levels of the messengers for COX-2, iNOS, MMP-13, RUNX-2 and VEGF, but to a different degree and with variable responses as a function of the maturation stage of micromasses. In particular, the chemokine proved to be a better inducer of COX-2 and VEGF in fresh micromasses, and of iNOS, MMP-13 and RUNX-2 in older constructs. Notably, HT pre-treatment prevented these increases for all markers and at different ages of micromasses. We have also considered the expression of SIRT-1 mRNA and found a different pattern with respects to the previous genes, with a low content of the mRNA after GROα and an increase following co-treatment with HT and GROα, which resulted significant in 1- and 2-week old micromasses compared to GROα alone or control cells (Figure 6F). Therefore HT was able to modulate gene expression in micromasses in a favourable way to counteract the appearance of a OA chondrocyte phenotype.

## Discussion

The”Mediterranean diet”, characterized among other features by a high consumption of olives and olive oil, has been associated with a healthy lifestyle and a lowered incidence of chronic diseases. Most of studies have focused on cardiovascular diseases, cancer and diabetes, however recently dietary supplementation of extra-virgin olive oil together with mild physical activity has been reported to prevent cartilage degeneration in a OA rat model [31]. Although initial reports attributed the beneficial properties of olive oil to its composition of fatty acids with the high content of oleic acid, accumulating evidence now indicates that its minor components, particularly polyphenols, critically contribute to these health benefits. In particular, HT has attracted much attention because of its relative abundance in olive products, its safety profile and its orthodiphenolic structure, which is related to its notable amphipatic behaviour, bioavailability and anti-oxidant efficacy [13]. It has also been found that olive vegetation water (OVW), a by-product of olive extraction rich in phenolics, acted synergistically with glucosamine to reduce serum TNF-α levels in lipopolysacharide (LPS)-treated mice, suggesting, according to Authors' words, that a combination of OVW and glucosamine may be an effective therapy for a variety of inflammatory processes [32]. A further report by the same group actually showed that OVW treatment decreases pain and improves daily activities of patients with OA and rheumatoid arthritis (RA) [33]. Interestingly, administration of oleuropein aglycone, an olive polyphenol secoiridoid, which can be partially hydrolyzed to HT, proved to exert an anti-inflammatory effect and ameliorate the tissue damage in mice with type II collagen-induced arthritis [34], an experimental model of RA. In addition, oleocanthal, a further phenolic compound identified in extra-virgin olive oil, is purported to have ibuprofene-like effects and has been shown to inhibit the production of pro-inflammatory factors, such as NO, iNOS, MIP-1α and IL-6, in a murine chondrocyte cell line [35], [36]. Thus it is possible that various phenolics and other components of olives and manufacturing derivatives may act together, even synergistically, to provide protective outcomes on various forms of arthritis. It should also be noted that HT and related phenolics may be present in significant amounts even in white wine and contribute to its beneficial effects [37], [38].

In the present study we have found that HT protects human chondrocytes exposed to significative pathogenic stimuli, i.e. H_2_O_2_ and GROα. In particular HT reduced or prevented increases in cell death and activation of executive caspases, DNA damage, expression of pro-inflammatory genes (COX-2, iNOS) and of genes driving chondrocyte terminal differentiation (RUNX-2, MMP-13 and VEGF), all of which characterizing OA pathogenesis. Particularly significant is the protective effect afforded versus GROα in 3-D micromass cultures, a useful *in vitro* model of OA. In fact we have previously showed that chondrocytes re-differentiate in micromasses and after 1 week recover an “healthy articular chondrocyte phenotype” being able to produce an abundant ECM rich in collagen 2 and aggrecan, the main ECM components of healthy articular cartilage. Afterwards this 3-D cartilaginous construct recapitulates the chondrogenesis process driven by ECM remodelling, so that at 3 weeks features of “hypertrophy, terminal differentiation, calcification and cell death” are observed [39], [40]. This model has been successfully employed to define the critical role of MMP-13 in concert with other regulatory factors [39], [40].

H_2_O_2_ and less markedly GROα were proved to increase the level of ROS in chondrocytes, which was reduced by pre-treatment with HT. The cytoprotective and anti-oxidant actions of HT, shown in a variety of cell types, have been related not only to its free radical scavenging activity, but also to the ability to enhance the endogenous defence system by inducing antioxidant/detoxifying enzymes through activation of transcription factors such as Nrf2 [14], [23] or FOXO3a [15]. Interestingly HT, differently from other olive oil phenolics tested, significantly increased transactivation in a Nrf2 gene reporter experiment with NIH 3T3 cells [41]. In addition HT has been shown to inhibit NF-κB activation in phorbol ester-treated vascular endothelial cells [22] and in LPS-treated THP-1 cells [17]. In its turn NF-κB inhibition can keep down-regulated pro-inflammatory and catabolic genes, such as those coding for iNOS, COX-2 and MMPs [18], [20]. It should be noted that these genes are regulated mainly at transcriptional level by pro-inflammatory pathways, therefore our finding of a reduced expression of their mRNAs supports a HT effect in antagonizing the activation of pro-inflammatory pathways like NF-κB even in chondrocytes.

Another potential mediator of the protective effect exerted by dietary polyphenols is SIRT-1, a NAD^+^-dependent protein deacetylase, which can be beneficial by modulating oxidative stress, inflammation, cellular senescence, apoptosis, autophagy, metabolism and mitochondrial biogenesis [42]. HT has been reported to induce the expression of SIRT-1 in rat hearts subjected to ischemia/reperfusion [38]. An enhanced SIRT-1 expression has been observed in breast cancer cells treated with secoiridoid polyphenols extracted from extra-virgin olive oil [43] and in the heart of SAMP8 mice fed with a diet enriched with olive oil phenolics [41]. In the present paper we show that HT enhanced SIRT-1 mRNA expression in GROα-stimulated micromasses, suggesting a role for this “longevity factor” in the HT protective action. Although HT did not increase significantly the level of SIRT-1 mRNA in H_2_O_2_-stimulated chondrocytes in monolayer, an involvement of SIRT-1 in protection from H_2_O_2_ cannot be excluded, since SIRT-1 expression and activity can be regulated at different levels, including by post-translational modifications [29], [42], [44].

In conclusion this study is the first to show a wide range of HT effects protecting human chondrocytes under *in vitro* conditions simulating OA. The precise mode of action of HT and the signaling pathways involved remain to define, nevertheless the use of HT to treat or prevent OA is supported by our results, and deserves further studies in animal models and clinical settings.
